# Factors associated with food safety practices among food handlers: facility-based cross-sectional study

**DOI:** 10.1186/s13104-019-4702-5

**Published:** 2019-10-22

**Authors:** Jember Azanaw, Mulat Gebrehiwot, Henok Dagne

**Affiliations:** Department of Environmental and Occupational Health and Safety, Institute of Public Health, College of Medicine and Health Sciences, University of Gondar, Gondar, Ethiopia

**Keywords:** Food handlers, Food safety, Hygiene practices, Food establishment, Food-borne diseases, Ethiopia

## Abstract

**Objective:**

The primary objective of this study was to assess factors associated with food safety practices among food handlers in Gondar city food and drinking establishments. The facility-based cross-sectional study was undertaken from March 3 to May 28, 2018, in Gondar city. Simple random sampling method was used to select both establishments and the food handlers. The data were collected through face-to-face interview using pre-tested Amharic version of the questionnaire. Data were entered and coded into Epi info version 7.0.0 and exported to SPSS version 22 for analysis.

**Results:**

One hundred and eighty-eight (49.0%) had good food handling practice out of three hundred and eighty-four food handlers. Marital status (AOR: 0.36, 95% CI 0.05, 0.85), safety training (AOR: 4.01, 95% CI 2.71, 9.77), supervision by health professionals (AOR: 4.10, 95% CI 1.71, 9.77), routine medical checkup (AOR: 8.80, 95% CI 5.04, 15.36), and mean knowledge (AOR: 2.92, 95% CI 1.38, 4.12) were the factors significantly associated with food handling practices. The owners, managers and local health professionals should work on food safety practices improvement.

## Introduction

Food safety continues as a critical problem in developed and developing nations for people, food companies and food control officials [1, 2]. Food-borne diseases (FBD) are associated with outbreaks and threatens global public health security and has got an international concern [3]. Food safety is a growing public health issue [4]. FBD is responsible for significant morbidity and mortality rates [5]. The worldwide incidence and financial expenses of food-borne diseases are hard to determine [6]. However, reports estimate that 2.1 million individuals died each year as a result of foodborne disease [5].

According to the WHO, FBDs in developing nations are serious because of bad hygienic food handling methods, bad understanding and absence of infrastructure [7]. This is due to the prevailing poor food handling and sanitation practices, inadequate food safety laws, weak regulatory systems, lack of financial resources, etc. [6, 8]. Evidence revealed that around 70% of diarrhoea cases were attributed to food-borne routes in developing countries [6]. Like other developing countries, the burden of food-borne diseases is growing in Ethiopia [18].

Approximately 10 to 20% of FBD outbreaks are because of contamination due to poor food handling practice of the food handlers [9]. In the absence of well-maintained and proper food handling practices in mass catering establishments have the potential to impart a disastrous effect on human health [6, 11].

Good personal hygiene and food handling practices are important for preventing the transmission of pathogens from food handlers to the consumers [12–14]. Close to 75% of food-borne illness outbreaks are attributed to lack of safe food handling practices by food handlers in food service establishments [5]. Food handlers play a key role in ensuring strict adherence to food safety principles throughout the whole process [15].

There is a high expansion of food establishments observed in the country including Gondar city. But ensuring safe food service has been one of the major challenges and concerns for producers, consumers and public health officials. Studies revealed that lack of basic sanitary facilities/infrastructures, poor knowledge and practice of hygiene and sanitation among food handlers in food service establishments, and negligence in safe food handling are major reasons of poor food safety practice in food establishments [16, 17]. Therefore, it is very essential to identify factors affecting safe food handling practices, especially during preparation and serving. Thus, this study aimed to evaluate factors associated with food safety practice among food handlers in Gondar city food establishments.

## Main text

### Methods

This facility-based cross-sectional study was conducted from March 3 to May 28, 2018 at Gondar city. Gondar city is one of the highly populated cities in northwest Ethiopia. There were a total of 326 food establishments and 4232 food handlers in Gondar city according to tourism office data. The city is found at 738 km away from Addis Ababa the capital city of Ethiopia. Ninety-eight food establishments were included using the rule of thumb by taking 30% of the total food establishments. n = N × 30% = 326 × 30/100 = 97.8 ≈ 98 none star food establishments.

The sample size was computed using a single population proportion formula with 95% CI, 5% marginal error (d) and p = 52% proportion of food handlers having good food handling practice from the previous study [19]. Based on these assumptions, 384 food handlers were included in the study.

To select food establishments and food handlers, a simple random sampling technique was used. In each institution, four food handlers were interviewed. After adaptation from similar literature [12, 19–21], the questionnaire was first prepared in English and translated to local language Amharic version. The pre-test was performed on 5% food handlers out of the study area before actual data collection. Then, correction and modification were undertaken based on the gaps identified during the pre-test. Reliability of the questionnaire was also evaluated. The information was gathered via a face-to-face interview using the questionnaire’s Amharic version. Four Environmental Health Officers have been engaged as data collectors and the principal investigator was involved as a supervisor. Food safety practice was the dependent variable in this research. Socio-demographic variables and behavioural factors were the independent variables. Food handling practice: food handlers were asked seventeen questions and those who scored less than or equal to the mean value were considered as having poor practice and those who scored greater than the mean value were considered as having good practice [19, 21]. Knowledge: Respondents were asked ten questions and those who scored less than or equal to the mean value were considered as having a poor knowledge [12, 22].

Consistency and completeness of data were verified during collection, entry and analysis. Data were entered and coded into version 7.0.0 of Epi Info and exported for evaluation to version 22 of SPSS. The data were analysed using descriptive (frequency and proportion), bivariate, and multivariable regression analysis. Variables with p-value < 0.25 during bivariate analysis were included in multivariable regression to assess the independent effect after controlling other variables [23].

We did Hosmer and Lemeshow test to check the model fitness. SPSS Cronbach’s Alpha test result for practice questionnaire was 0.83. Finally, 95% confidence level, AOR and p-value less than 0.05 were considered for determining statistically significant variables.

### Results

#### Sociodemographic characteristics of study participants

Of the three hundred eighty-four food handlers, 338 (88%) were females, 300 (78.1%) were unmarried; and 318 (82.8%) had an income of 500–1000 Ethiopian birr (28 ETB = 1 USD) (Table 1).

**Table 1:** Socio-demographic profile of food handlers in Gondar city food establishments, 2018 (n = 384)

Variables	Frequency (n)	Percentage (%)
Sex		
Male	46	12.0
Female	338	88.0
Age		
18–29	340	88.5
30–40	39	10.2
Above 40	5	1.3
Marital status		
Single	300	78.1
Married	63	16.4
Divorced	16	4.2
Widowed	5	1.3
Educational status		
Not read and write	37	9.6
Read and write	30	7.8
Primary school (1–8)	112	29.2
Secondary (9–10)	142	37.0
Higher education	63	16.4
Experience		
< 1 years	134	34.9
1–4 years	102	26.6
> 4 years	148	38.5
Level of income		
500–1000	318	82.8
1001–1500	32	8.3
> 1500	34	8.9

#### Knowledge of food handlers regarding the cause of food-borne disease, mode of transmission and way of food contamination

Three hundred sixteen (82.29%) of food handlers stated that food-borne diseases are caused by germs. More than half 199 (51.8%) of food handlers found this information from health center about food safety practices (Table 2).

**Table 2:** Knowledge of food handlers regarding food-borne disease, mode of transmission and way of food contamination in food establishments in Gondar city, 2018 (n = 384)

Variable	Frequency (n)	Percentage (%)
Source of information about food borne disease		
Mass media	113	29.4
Health professionals during the inspection	72	18.8
Health center	199	51.8
Cause of food borne disease		
Germs	316	82.3
Chemicals	43	11.2
Do not know	25	6.51
Route of transmission for food borne disease		
Dirty hand	343	89.3
Infected food handler	277	72.1
Dirty utensils	263	68.5
Vectors	194	50.5
Dirty work environment	182	47.4
Do not know	3	0.8
The critical times for hand washing		
After using the toilet	232	60.4
Before and after food preparation	333	86.7
After touching anything	174	45.3
After counting money	143	37.2
Route of transmission of food borne disease		
Contaminated food	341	88.8
Contaminated water	253	65.9
Vectors	172	44.8
Do not know	9	2.4
Ways of food contamination		
Dirty hand	343	89.3
Infected food handlers	277	72.1
Dirty utensils	263	68.5
Presence of vectors and rats	194	50.5
Dirty working environment	182	47.4
Symptoms of food borne disease		
Vomiting	162	42.2
Fever	162	42.2
Diaorrhea	294	76.6
Do not know	12	3.1
Germs found on cutting board		
Yes	335	87.2
No	49	
The food handlers perform correct procedures of washing food utensils	56	14.6
Food handlers properly use single knife	50	13.0
Total knowledge		
Poor	214	55.7
Good	170	44.3

#### Food handling practice of food handlers in food and drinking establishments

More than half of (51.5%) food handlers use hair net during food preparation. One hundred ninety (49.5%) of food handlers did not attend routine medical checkups. About 37% of the respondents were not wearing a uniform during handling and preparation of food (Table 3).

**Table 3:** Determinants of food safety practice among food handlers working in food and drinking establishments in Gondar City, 2018 (n = 384)

Variables	Food safety practice		COR (95% CI)	AOR (95% CI)
	Good	Poor		
Sex				
Male	21	25	0.86 (0.46, 1.60)	0.62 (0.26, 1.50)
Female	167	171	1	1
Marital status				
Single	142	158	0.36 (0.14, 0.95)	0.36 (0.05, 0.85)*
Married	31	32	0.08 (0.13, 1.13)	0.34 (0.08, 1.54)
Divorced	15	6	1	1
Educational status				
Not read and write	18	19	0.14 (0.02, 1.21)	0.45 (0.16, 1.33)
Read and write	15	15	0.14 (0.02, 1.31)	0.54 (0.16, 1.77)
Primary education	46	66	0.10 (0.01, 1.84)	0.51 (0.23, 1.14)
Secondary education	71	71	0.20 (0.02, 1.93)	0.88 (0.41, 1.91)
Higher education	38	25	1	1
Experience				
< 1 year	58	76	0.52 (0.42, 1.41)	0.71 (0.33, 1.53)
1–4 years	75	27	1.89 (0.28, 1.90)	0.80 (0.39, 1.65)
> 4 years	88	60	1	1
Level of income (ETB, 1 USD = 28 ETB)				
500–1000	160	158	1.01 (0.50, 2.05)	0.94 (0.36, 2.45)
1001–1500	11	21	0.52 (0.19, 1.41)	0.39 (0.11, 1.37)
> 1500	17	17	1	1
Food safety training				
Yes	79	30	4.01 (2.47, 6.51)	4.01 (2.71, 9.77) **
No	109	166	1	1
Supervision by health professionals				
Yes	106	47	4.09 (2.65, 6.34)	4.10 (1.71, 5.27) **
No	82	149	1	1
Level of knowledge				
Good	108	62	2.90 (1.92, 4.43)	2.92 (1.38, 4.12) *
Poor	80	134	1	1
Routine medical checkup				
Yes	142	52	8.55 (5.40, 13.5)	8.80 (5.04, 15.36) **
No	46	144	1	1

* Statistically significant at p < 0.05

** Statistically significant at p < 0.001 | Hosmer and Lemeshow test = 0.684 showed that the model fitted well

#### Factors associated with food safety practices

Multivariable logistic regression analysis revealed that marital status, food safety training, routine medical checkup, supervision by health professionals and knowledge were statistically associated variables with food safety practices.

Single food handlers were 64.0% less likely to practice food safety than the single food handlers (AOR: 0.36, 95% CI 0.05, 0.85). Food handlers supervised by health professionals were 4.10 times more likely to practice good food safety than non-supervised (AOR: 4.10, 95% CI 1.71, 5.27). Knowledgeable food handlers were 2.92 times more likely to practices good food safety than non-knowledgeable (AOR: 2.92, 95% CI 1.38, 4.12). Trained food handers were 4.01 times more likely to have good food handling practice than non-trained food handlers (AOR: 4.01, 95% CI 2.71, 9.77). Food handers followed routine medical checkup had 8.80 times more likely to have good food handling practice than not- followed food handlers (AOR: 8.80, 95% CI 5.04, 15.36) (Table 3).

### Discussion

One hundred eighty-eight (49.0%) food handlers had good food safety practice. This finding is lower than the findings of studies in Bahir Dar (67.6%) [24], Arba Minch (67.4%) [21] and in Dubai (81.74%) [17]. While the finding was close with studies in Dangila town (52.5%), Addis Ababa (52.3%), Imo State, Nigeria (50%) and Turkey (48.4%) [6, 19, 25, 26], respectively. However, it is higher than the studies done in Gondar town (22.1%) [5], South-Western Nigeria (19.0%) [27], Ogun, Nigeria (31.5%) [19]. These variations might be due to the difference in the study design, variation in training, and the provision of food hygiene and safety inputs. About 109 (28.4%) of the food handlers were certified in food safety training. This result is higher as compared with findings from Bahir Dar (21.8%) and Mekelle (15.7%) [12, 28]. Food handler training is seen as one strategy whereby food safety practice can be increased, offering long-term benefits to the food establishments [29]. This finding is supported with studies conducted India [10], Nigeria [30], Ghana [31] and Dubai [32]. The number of food handlers who recieved food safety training in the current study is higher than with findings from Bahir Dar (21.8%), and Mekelle (5.4%) [12, 28]. Food handlers who received training would have a better understanding of safe food handling practice as they might get professional advice during training. Training could enhance food handlers overall performance in safe food handling practice [21]. In this study, food handlers who got safety training had higher odds of good food safety practice. This might be due to trained food handlers gain good awareness through training. This supported with other similar study done in Sarawak [33]. Training programs are important for improving the knowledge of food handlers [34]. Food safety practice was also positively associated with the level of knowledge. The probability of having a good food safety practice among participants with good level of knowledge was 2.39 times higher with compared to those with a poor level knowledge (AOR = 2.39, 95% CI 1.38, 4.12). Food handlers are expected to have substantial knowledge and skills for handling foods hygienically [12]. This might be due to those food handlers who had a good level knowledge might have a higher chance of good food handling practice. This finding was supported studies conducted in Gondar town, and Malaysia [5, 15]. Marital status was another significantly associated factor with food safety practices. Single food handlers had lower probability of good food safety practices compared with divorced handlers. This is supported with the study done in Gondar town and Dangila town [19].

Food safety practice was significantly associated with supervision by health professionals. The probability of having good food safety practice was higher among food handlers supervised by health professionals as compared with non-supervised. This finding was supported by the study conducted in Arba Minch [21]. This might be due to supervisors give advice for food handlers, the owners and to the managers. A routine medical checkup was also another factor significantly associated with good food handling practice. The probability of having good food safety practice among food handlers engaged with routine medical checkup was higher than food handlers not engaged in routine medical checkup. This could be the health care workers gave advice for food handlers during examination. This finding is in line with studies conducted in Arba Minch and Dessie towns [20, 21]. This study revealed that there was poor food handling practice among food handlers. Marital status, food safety training, supervision by health professionals, routine medical checkup, and level of knowledge of food handlers were significantly associated with good food handling practice. Owners, managers and local health professionals should enhance the level of knowledge of food handlers, provide food hygiene, safety training, undertake periodic supervision, and routine medical checkup.

### Limitations

This study was not without limitations. Some of the limitations include inherent weakness of cross-sectional study to establish a cause–effect relationship, social desirability bias and recall bias.

## Data Availability

We will make data available upon request the primary author.

## Abbreviations

WHO: World Health Organization
OR: adjusted odds ratio
CI: confidence interval
COR: crude odds ratio
SPSS: Statistical Package for Social Sciences
ETB: Ethiopian Birr
IRB: Institutional Review Board
